# Effect of Cultivar on Chlorophyll Meter and Canopy Reflectance Measurements in Cucumber

**DOI:** 10.3390/s20020509

**Published:** 2020-01-16

**Authors:** Romina de Souza, Rafael Grasso, M. Teresa Peña-Fleitas, Marisa Gallardo, Rodney B. Thompson, Francisco M. Padilla

**Affiliations:** 1 Department of Agronomy, University of Almeria, Carretera de Sacramento s/n, La Cañada de San Urbano, 04120 Almería, Spain; mtpena.fl@ual.es (M.T.P.-F.); mgallard@ual.es (M.G.); rodney@ual.es (R.B.T.); f.padilla@ual.es (F.M.P.); 2 Estación Experimental INIA Salto Grande, Instituto Nacional de Investigación Agropecuaria (INIA), Camino al Terrible s/n, 50000 Salto, Uruguay; rgrasso@inia.org.uy; 3 CIAIMBITAL Research Centre for Mediterranean Intensive Agrosystems and Agrifood Biotechnology, University of Almeria, La Cañada de San Urbano, 04120 Almería, Spain

**Keywords:** genotype, greenhouse, leaf nitrogen, proximal optical sensors, vegetation index

## Abstract

Optical sensors can be used to assess crop N status to assist with N fertilizer management. Differences between cultivars may affect optical sensor measurement. Cultivar effects on measurements made with the SPAD-502 (Soil Plant Analysis Development) meter and the MC-100 (Chlorophyll Concentration Meter), and of several vegetation indices measured with the Crop Circle ACS470 canopy reflectance sensor, were assessed. A cucumber (*Cucumis sativus* L.) crop was grown in a greenhouse, with three cultivars. Each cultivar received three N treatments, of increasing N concentration, being deficient (N1), sufficient (N2) and excessive (N3). There were significant differences between cultivars in the measurements made with both chlorophyll meters, particularly when N supply was sufficient and excessive (N2 and N3 treatments, respectively). There were no consistent differences between cultivars in vegetation indices. Optical sensor measurements were strongly linearly related to leaf N content in each of the three cultivars. The lack of a consistent effect of cultivar on the relationship with leaf N content suggests that a unique equation to estimate leaf N content from vegetation indices can be applied to all three cultivars. Results of chlorophyll meter measurements suggest that care should be taken when using sufficiency values, determined for a particular cultivar

## 1. Introduction

In intensive vegetable production, large applications of nitrogen (N) fertilizer are used to ensure high yields [1,2]. The amounts of N applied often appreciably exceed crop requirements; the excess N is susceptible to nitrate (NO_3_^−^) leaching [2,3], and to subsequent N contamination of aquifers and surface water bodies [4,5]. Nitrate contamination of aquifers and surface water bodies, from intensive vegetable production, has been reported for diverse regions, such as southeast Spain [6], southeast United States [3] and China [1,7].

For optimal management of N in intensive crop and vegetable production, with minimal N loss to the environment, it is necessary to match N supply to crop N demand [8]. Assessment of crop N status informs of the immediate balance between N supply and demand [8,9]. An effective and rapid means to assess crop N status is through the use of proximal optical sensors [5,8,10]. Chlorophyll meters have been extensively researched and are used commercially to assess crop N status because their measurements of relative leaf chlorophyll content are generally strongly related to leaf N content, which reflects crop N status [5,10,11,12]. Chlorophyll meters make non-destructive measurements of relative leaf chlorophyll content by measuring the absorbance and transmittance of radiation of two light wavelengths by the leaf. Chlorophyll absorbs red radiation and transmits most of the near infra-red (NIR) radiation, which is influenced by leaf thickness, among several parameters [5,10,12]. Absorbance of red radiation increases with chlorophyll content, resulting in higher chlorophyll meter values [5,12]. Chlorophyll meters are well suited for on-farm use because they are easy to operate, do not require any particular training, and make measurements quickly [5,13]. Given these characteristics, chlorophyll meters are useful practical tools for assessing crop N status to identify required adjustments in N fertilizer application to ensure optical crop N status [5]. 

Canopy reflectance sensors can be used in commercial farming to determine crop N fertilizer requirements, and for variable rate N fertilizer application [10,11]. These sensors assess crop N status by measuring the reflection of two or more specific wavelengths of radiation from crop foliage [14]. Visible and near-infrared wavelengths are used [5,10]. The reflectance of the measured wavelengths is entered into mathematical equations to derive vegetation indices. Numerous vegetation indices are available, depending on the wavelengths and formula used. Vegetation indices have been reviewed by Bannari et al. [15], Ollinger [14] and Hatfield and Prueger [16], who described the appropriate applications of the various indices. The most widely-used vegetation index is the normalized difference vegetation index (NDVI) [5,17]. Proximal canopy reflectance sensors are a form of remote sensing in which sensors are placed close to the crop; the distance ranging from several centimeters to several meters from the canopy [5]. Reflectance sensors detect crop responses that are sensitive to crop N status, such as leaf chlorophyll, foliage greenness, foliage density and biomass [10]. The advantage of reflectance measurements is that they can integrate a substantially larger surface area of the crop than single leaf measurements made with a chlorophyll meter [8,12].

Considerable research has demonstrated the capacity of proximal optical sensors to assess crop N status in various field crops, mostly in cereals such as rice [18], maize [19] and wheat [20,21,22]. Additionally, their capacity to assess crop N status has been evaluated in diverse horticultural crops such as potato [23,24], tomato [25,26,27], cucumber [28] and muskmelon [29]. Most of the research with proximal optical sensors to assess crop N status has been with a specific cultivar of a given species. Few reports have examined how differences between cultivars affect optical sensor measurements.

Working with wheat, Monostori et al. [30], reported that cultivar had a notable effect on the relationship between chlorophyll meter (SPAD-502) readings and grain yield. Similar results with wheat were obtained by Hoel [31] using the Hydro N-Tester chlorophyll meter. In rice, the relationship between SPAD-502 measurements and leaf N content differed markedly with genotype [32]. In tomato, Sandoval-Villa et al. [33] reported significant differences in chlorophyll meter measurements in one cultivar compared to four others, but not amongst the other four cultivars. 

Few studies have examined how cultivar influences measurements made with canopy reflectance sensors; the few reported studies have examined only the NDVI index. The NDVI was able to differentiate different cultivars at different growth stages in wheat [17]. With wheat also, Samborski et al. [34] obtained statistically significant differences in NDVI between cultivars in one growth stage. Available reports suggest that cultivar effects on reflectance measurements can occur in cereal crops. We are unaware of published relevant information for vegetable crops. 

Understanding cultivar effects on optical sensors such as chlorophyll meters and canopy reflectance sensors is fundamental for the use of these sensors in commercial farming. New cultivars are continually being introduced into commercial production; sometimes, there are notable phenotypic differences between cultivars. For a given species, it is necessary to identify if and to what extent cultivar affects optical sensor measurement. Secondly, if such effects are appreciable, procedures will need to be developed to deal with them when using optical sensors for crop N management.

The objectives of the present work were (1) to evaluate the effects of cucumber cultivar on chlorophyll meter measurements and vegetation indices measured with a canopy reflectance sensor, and (2) to assess how differences in cultivars affect the relationship between leaf N content and optical sensors measurements. Optical sensors measurements and their relationships with leaf N content were compared for three cucumber cultivars grown in a greenhouse, with three different N treatments.

## 2. Materials and Methods

### 2.1. Experimental Site

A cucumber (*Cucumis sativus* L.) crop was grown in soil in a greenhouse in conditions very similar to those of commercial greenhouse vegetable production in southeast (SE) Spain. The crop was grown in a multi-tunnel greenhouse at the Experimental Station of the University of Almería, located in Retamar, Almería, SE Spain (36°51′51″N, 2°16′56″W and 92 m elevation; a detailed description of the greenhouse is provided by Padilla et al. [28]. The crop was grown in an “enarenado” soil typical of those used for soil-grown greenhouse production in Almería. More information on the soil used is provided by Padilla et al. [29]. A general description of “enarenado” soil is given by Thompson et al. [2].

The cropping area was 1300 m^2^, the crop rows were aligned north–south in paired lines. The greenhouse was divided in 12 plots of 12 m × 6 m each. Each plot contained six paired lines of plants, with 24 plants per line; the distance between plants in each line was 0.5 m. Separation between lines within a paired line was 0.8 m and the distance between adjacent paired lines was 1.2 m, giving a plant density of 2 plants m^−2^ and 144 plants per replicate plot. Sheets of polyethylene film (250 µm thickness) buried to 30 cm depth acted as a hydraulic barrier between plots [35].

Above-ground drip irrigation was used. There was one emitter per plant, each emitter had a discharge rate of 3 L h^−1^. All mineral fertilizer was applied through the drip irrigation system by fertigation. Complete nutrient solution was applied in each irrigation. Irrigation/fertigation occurred every 1–2 days depending on crop demand. 

### 2.2. Experimental Design

The experiment was carried out in 2018, the crop was transplanted on 24 April and ended on 3 July, being grown for 70 days after transplanting (DAT). The crop was transplanted as 21-day old seedlings.

Three different cucumber cultivars, ‘Strategos’ (Syngenta International AG, Basel, Switzerland), ‘Pradera’ (Rijk Zwaan Zaadteelt en Zaadhandel B.V., De Lier, The Netherlands) and ‘Mitre’ (Semillas Fitó, Barcelona, Spain) were grown. The three cultivars were planted in each experimental plot, with one paired line (i.e., two lines per plot) of plants being planted with each cultivar. In each plot, there were three paired lines, one of each cultivar. The position of the paired lines of each cultivar in each plot was randomized.

There were three different N treatments that were applied to each of the cultivars. The N treatments were applied as different N concentration in the nutrient solution applied by fertigation. There were four replicated plots per treatment. The plots were organized in a randomized block design. The intended N treatments were very deficient (N1), sufficient (N2) and excessive (N3). 

Before transplanting, a series of large irrigations were applied, in total 402 mm, to leach residual NO_3_^−^ from the soil root zone and to homogenize the soil within the different plots. At the moment of transplanting, the mean soil mineral N content in the 0–60 cm depth (excluding gravel mulch) was 24, 34 and 63 kg N ha^−^^1^ in the N1, N2 and N3 treatments, respectively.

The average mineral N (N–NO_3_^−^ + N–NH_4_^+^) concentrations applied in the nutrient solution were 2.4, 8.5 and 14.8 mmol L^−1^, for the deficient, sufficient and excessive N treatments, respectively. During the first four days after transplanting, the plants were irrigated with water only (0.1 mmol N L^−1^) and during the next four days, all three treatments received a common nutrient solution of 1.0 mmol N L^−1^. Differential N treatments began nine days after transplanting and continued until the end of the crop. Regardless of the treatment, most N was applied as a NO_3_^−^ (91% of applied N) and the rest as NH_4_^+^. All other nutrients were applied in the nutrient solution to ensure they were not limiting.

General crop management followed standard local practice; the crops were periodically pruned and were supported by nylon cord guides. Irrigation was scheduled to maintain the soil matric potential (SMP) in the root zone, at 15 cm depth between −10 and −30 kPa. One tensiometer (Irrometer, Co., Riverside, CA, USA) was installed in each plot to measure SMP [35]. Topping (the removal of the apical shoot to arrest stem elongation) was conducted on 46 DAT.

### 2.3. Optical Sensors Measurements

Optical measurements of relative leaf chlorophyll content were made with two hand-held leaf-clip sensors, the SPAD-502 (Minolta Camera Co. Ltd., Tokyo, Japan) and the MC-100 Chlorophyll Concentration Meter (Apogee Instruments, Inc., Logan, UT, USA). For individual measurements, the SPAD-502 measures a leaf surface area of 6 mm^2^ and the MC-100 an area of 63.6 mm^2^. The SPAD-502 measures absorbance at 650 nm (red) and 940 nm (NIR), and the MC-100 at 653 nm and 931 nm. Measurements with both sensors were made by clipping the sensor onto the leaf. 

Measurements with chlorophyll meters commenced at 22 DAT. Measurements were then made weekly until the end of the crop and were made on seven dates. Measurements were made on each of eight marked plants, of each cultivar, in each replicate plot. They were made at the same time (8:00–10:00 solar time), before irrigation/fertigation was applied. On each plant on each measurement date, one measurement was made on the most recently fully expanded and well-lit leaf, on the distal part of the adaxial side of the leaf, midway between the margin and the mid-rib of the leaf, consistent with the protocol developed by Padilla et al. [29,36]. Leaves with physical damage or with condensed water were not measured; alternative plants being selected. After topping and the associated cessation of new leaf production, measurements were made on the same leaf of the selected plants [29].

Measurements of canopy reflectance were made with the Crop Circle ACS-470 sensor (Holland Scientific Inc., Lincoln, NE, USA), which is an active proximal canopy reflectance sensor [37]. Filters were selected to measure reflectance at 550 nm (green), 670 nm (red) and 760 nm (near-infrared, NIR). The sensor was held vertically parallel to the crop rows, facing the upper part of the foliage at a 45 cm horizontal distance giving a field of view on the foliage surface of 26 cm (height) × 5 cm (width) [29]. The sensor was positioned so that the top of the field of view was level with the most recently fully expanded leaf, in accordance with the protocol developed by Padilla et al. [27,29] in greenhouse-grown vertically supported crops. Measurements were always made at the same time each day (10:00–11:00 solar time). They commenced once the crop had sufficient height to enable measurement considering the 26 cm height of the field of view, at 29 DAT. Measurements continued weekly until the end of the crop, for a total of six measurement dates. In each replicate plot, four measurement passes of 4 m were made, for each cultivar, at walking speed (approx. at 1.5 km h^−1^). There were ten measurements per second, giving approximately 400 individual measurements per plot. Reflectance data of each wavelength were stored in a portable GeoScout GLS-400 data logger (Holland Scientific, Inc., Lincoln, NE, USA) and subsequently processed.

From each individual reading, four vegetation indices were calculated based on the reflectance values of individual wavelengths. The individual index values from each reading were then averaged to provide a single value for the measurement in each replicate plot. The indices were: (i) normalized difference vegetation index (NDVI) [38], (ii) the normalized difference vegetation index on greenness (GNDVI) [39], which is a variation on NDVI using the green wavelength, (iii) the red ratio vegetation index (RVI) [40] and (iv) green ratio vegetation index (GVI) [40]. These indices are among the reflectance indices of vegetation most commonly used to evaluate crop N status [5,10,11,41,42].

### 2.4. Leaf N Content

On each date of measurement with optical sensors, eight plants per cultivar and replicate plot were selected, and the most recently fully expanded leaf was removed for determination of total N content (%N). Measurement of leaf N content is a long established method for assessment of crop N status of vegetable crops [8]. The removed leaves were placed in a paper bag and oven dried at 65 °C until constant weight. Petioles were discarded. Dry material was ground sequentially in knife and ball mills. The total N content (%N) of each sample was determined using a Dumas-type elemental analyzer system (model Rapid N, Elementar, Analysen systeme GmbH, Hanau, Germany).

### 2.5. Cultivar Characterization

To characterize the three cultivars, measurements of crop height (level of the gravel mulch to top leaf) were made immediately before topping, at 46 DAT, in eight plants per cultivar. Leaf color analysis was performed on eight of the latest fully expanded leaves of each cultivar in each replicate plot. A colorimeter (Minolta Chroma Meter CR-400, Konica Minolta, Osaka, Japan) was used, providing CIE 1931 color space coordinates (i.e., luminance (Y), chromatic coordinate x and chromatic coordinate y). For determination of leaf area index (LAI), a destructive sampling was conducted in which all leaves from a randomly selected plant per cultivar and replicate plot were removed at 45 DAT. After excision, leaves were kept refrigerated in zip-lock plastic bags and immediately taken to the laboratory. Total leaf area was measured with an area meter (LI-3100C; LI-COR, Inc., Lincoln, NE, USA). LAI was calculated by dividing total leaf area by sampled soil area.

### 2.6. Statistical Analysis

For measurements conducted one time during the crop, such as LAI, crop height and leaf color, factorial analysis of variance (ANOVA) was performed to the test the effects of N treatments and cultivars on the measured variables. For measurements taken several times during the crop, such as leaf N content and optical sensor measurements, repeated-measure analysis of variance (RM-ANOVA) were conducted to test the effects of N treatments, cultivars and time on measured variables. Homogeneity of variances was checked prior to ANOVA analysis and variables were transformed if ANOVA assumptions were not met. The IBM SPSS 25 software program (IBM Corporation, Armonk, NY, USA) was used.

Linear regressions between leaf N content (dependent variable) and optical sensor measurement (independent variable) were evaluated for each cultivar and date of measurement separately. Coefficient of determination (R^2^), standard error of the estimate (SSE), probability (*p*-value), slope and intercept, were calculated using the IBM SPSS 25 software. 

To compare the effect of cultivar on the relationship between leaf N content and optical sensor measurement, the methodology used by ArchMiller et al. [43] was used. Firstly, the relationship between leaf N content and optical sensor measurement for the three cultivars together was established, for chlorophyll and canopy reflectance sensor measurements. This regression equation was called “reduced regression”:

(1) Leaf N content=a + b x (Optical sensor measurement),

where a and b are the intercept and slope of the regression, respectively. Secondly, the change in linear regression between leaf N content and optical sensor measurement of the reduced regression calculated in Equation (1), and linear regression between leaf N content and optical sensor measurement of each of the three cultivars separately, was analyzed with the sum of squares reduction test (F-statistic), for each date of measurement, using the equation:

(2) $$F-\mathrm{statistic}=\frac{\left(\mathrm{SSEred}-\mathrm{SSEcultivar}\right)/\left(\mathrm{dfred}-\mathrm{dfcultivar}\right)}{\mathrm{SSEcultivar}/\mathrm{dfcultivar}},$$

where SSEred and SSEcultivar are the error sum of squares and dfred and dfcultivar are the degrees of freedom, of the reduced and each cultivar regression, respectively. Each cultivar regression had individual a and b parameters. To analyze if the reduced regression was different from the cultivar regression, the F-statistic was used to calculate the *p*-value. *p*-values ≤ 0.05 indicate that the reduced regression was statistically different from the cultivar regression, thus indicating a significant effect on cultivar on the relationship between leaf N content and optical sensor measurements.

## 3. Results

### 3.1. Cultivars Characterization

Crop height was not significantly different between cultivars (*p* > 0.05). However, there were statistical differences between cultivars in LAI, luminance and chromatic coordinates x,y (*p* < 0.05) (Table S1 and Figure S1); ‘Strategos’ had significantly higher LAI, luminance and x,y coordinate values than ‘Pradera’ and ‘Mitre’ (Table 1).

### 3.2. Differences in Leaf N Content between Cultivars

**Figure 1:**
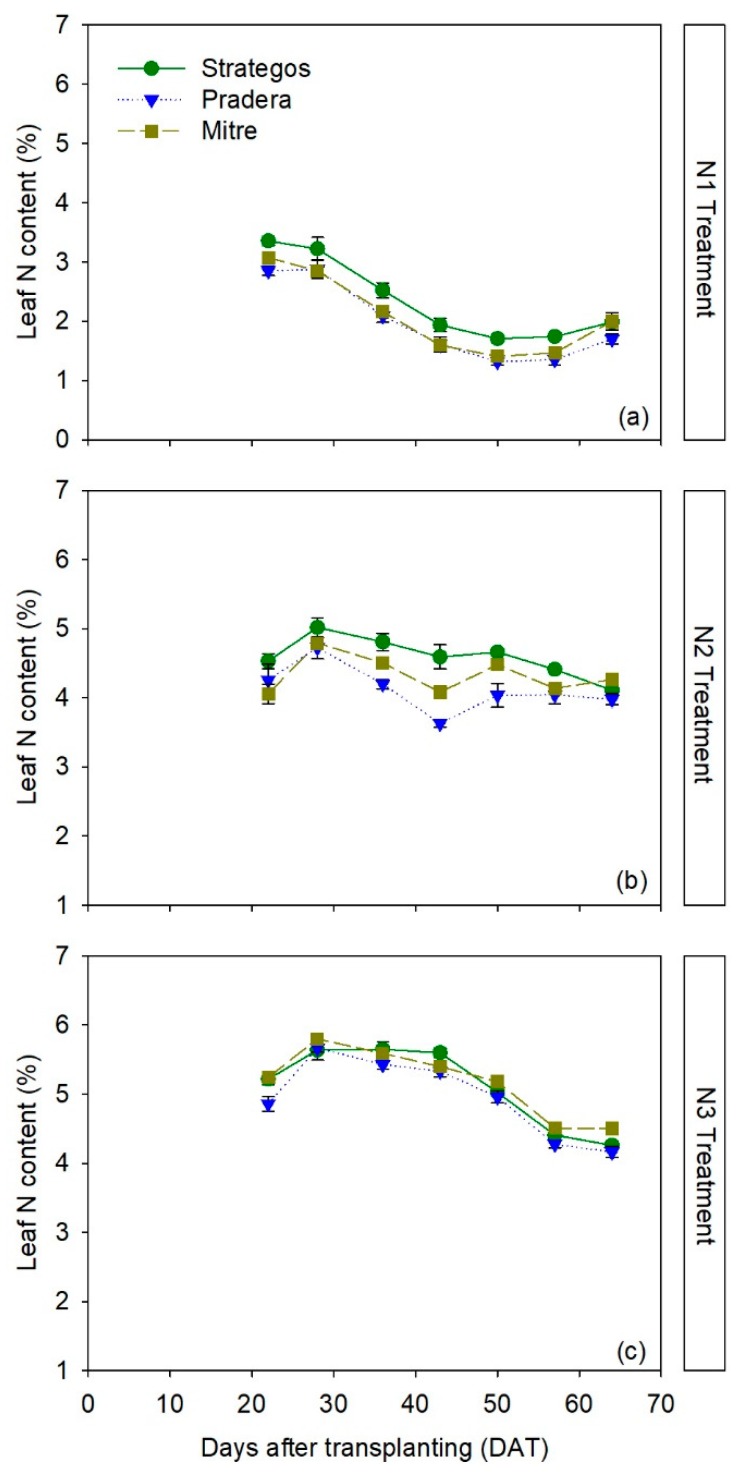
Temporal dynamics of leaf N content (%) of three cultivars of cucumber (*Cucumis sativus* L. ‘Strategos’, ‘Pradera’ and ‘Mitre’) under three N treatments (N1 (panel **a**), N2 (panel **b**) and N3 (panel **c**)). Values are means ± SE.

There were significant differences between cultivars in leaf N content values depending on N treatment and time (RM-ANOVA, *p* < 0.05; Table S2). In the N1 treatment, ‘Strategos’ had significantly higher leaf N content than ‘Pradera’ and ‘Mitre’ throughout most of the crop. ‘Pradera’ had the lowest leaf N content, but it was not significantly lower than ‘Mitre’ (Figure 1a). Average leaf N content in the N1 treatment for the whole crop cycle was 2.35% ± 0.05%, 2.08% ± 0.04% and 1.97% ± 0.09%, for ‘Strategos’, ‘Mitre’ and ‘Pradera’, respectively. 

In the N2 treatment, ‘Strategos’ had the highest leaf N content, ‘Pradera’ the lowest and ‘Mitre’ had an intermediate leaf N content (Figure 1b). Average leaf N contents for the N2 treatment for whole crop cycle were 4.59% ± 0.07%, 4.33% ± 0.04% and 4.12% ± 0.10%, for ‘Strategos’, ‘Mitre’ and ‘Pradera’, respectively. 

In the N3 treatment, there were no clear differences between cultivars in leaf N content (Figure 1c). Average leaf N content in the N3 treatment for the whole crop was 5.11% ± 0.05%, 5.17% ± 0.03% and 4.95% ± 0.05%, for ‘Strategos’, ‘Mitre’ and ‘Pradera’, respectively.

### 3.3. Chlorophyll Meter Measurements

The RM-ANOVA indicated significant differences between cultivars in chlorophyll meter measurements, depending on N treatment and time, both for the SPAD-502 meter (RM-ANOVA, *p* < 0.001) and for the MC-100 meter (RM-ANOVA, *p* < 0.001; Table S3). Generally, in all treatments ‘Mitre’ was the cultivar with the highest SPAD values, ‘Strategos’ had the lowest SPAD values, and ‘Pradera’ was intermediate. The average differences in SPAD values throughout the crop, considering the three N treatments, were the following: ‘Mitre’ was 3.7 ± 1.0 SPAD units higher than ‘Pradera’, and ‘Pradera’ was 2.6 ± 1.1 SPAD units higher than ‘Strategos’. Expressed as percentages, these differences were 8.1% and 6.2%, respectively.

**Figure 2:**
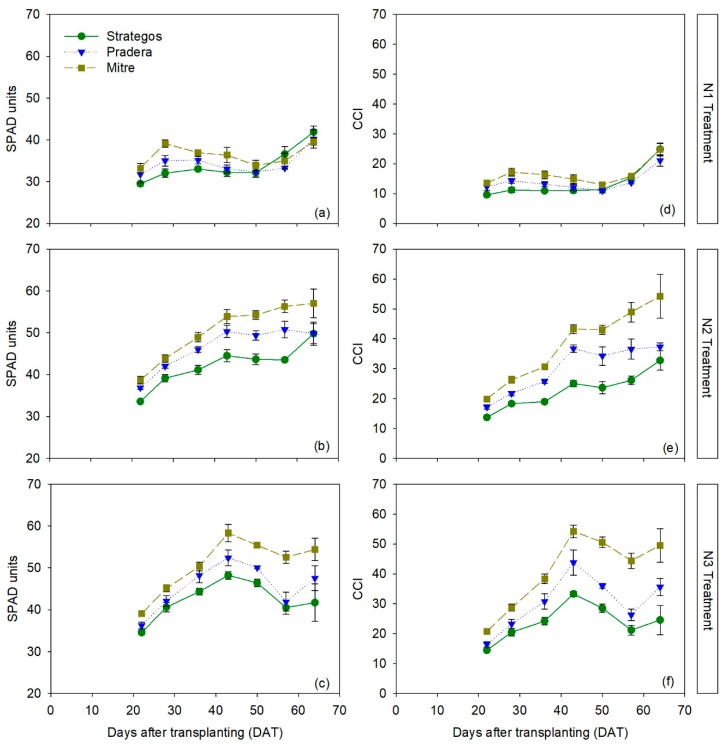
Temporal dynamics of SPAD (panels **a**–**c**) and chlorophyll content index (CCI) measurements (panels **d**–**f**) of three cultivars of cucumber (*Cucumis sativus* L. ‘Strategos’, ‘Pradera’ and ‘Mitre’) under three N treatments (N1, N2 and N3). Values are means ± SE.

For the N1 treatment, there were no significant differences between the three cultivars throughout the crop (Figure 2a). In the N2 and N3 treatments, SPAD values of ‘Mitre’ were statistically significantly higher than those of ‘Pradera’ and ‘Strategos’. In N2 treatment, SPAD values of ‘Pradera’ were consistently statistically higher than those of ‘Strategos’ (Figure 2b). 

For measurements with the MC-100 meter, ‘Mitre’ had significantly higher chlorophyll content index (CCI) values than ‘Pradera’ and ‘Strategos’ in the N2 and N3 treatments (Figure 2e,f). In the N1 treatment, there were no statistical differences (Figure 2c). For each of the three N treatments, ‘Mitre’ had the highest CCI values, ‘Strategos’ the lowest and ‘Pradera’ was intermediate. Averaged throughout the crop and for the three N treatments, ‘Mitre’ had CCI values that were 7.1 ± 2.4 CCI units higher than ‘Pradera’ and ‘Pradera’ was 4.7 ± 2.2 CCI units higher than ‘Strategos’. In percentage terms, these values corresponded to differences of 22.3% and 19.1%, respectively.

### 3.4. Canopy Reflectance Measurements

**Figure 3:**
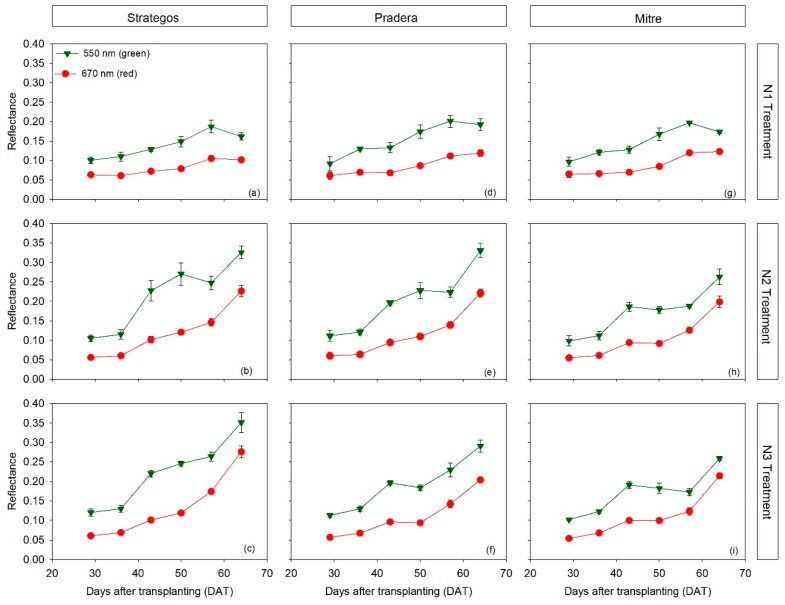
Temporal dynamics of red and green reflectance of three cultivars of cucumber (*Cucumis sativus* L. ‘Strategos’ (panels **a**–**c**), ‘Pradera’ (panels **d**–**f**) and ‘Mitre’ (panels **g**–**i**)) under three N treatments (N1, N2 and N3). Values are means ± SE.

There was a similar dynamics of red and green reflectance throughout most of the crop cycle, regardless of the N treatment (Figure 3). Reflectance of both red and green bands increased in the second half of the crop, particularly in N2 and N3 treatments (Figure 3).

**Figure 4:**
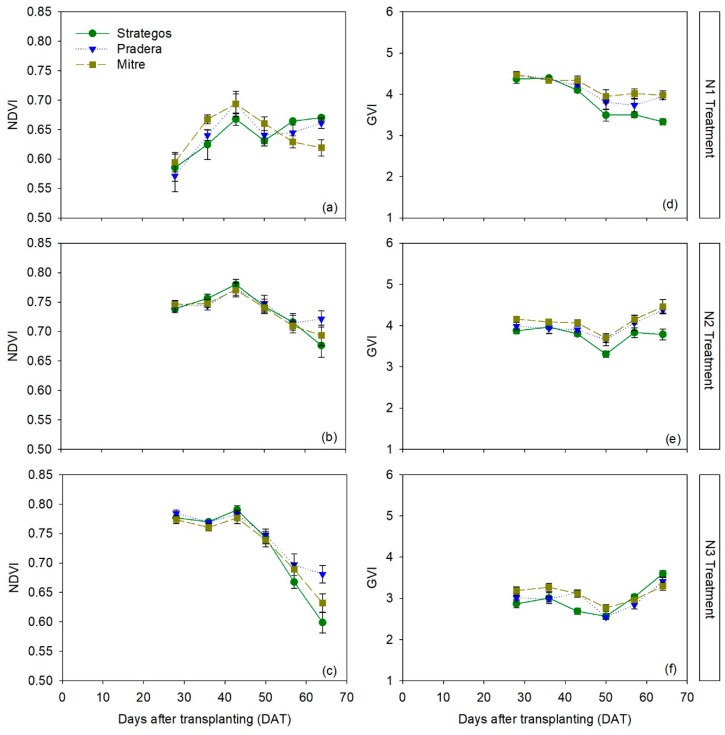
Temporal dynamics of normalized difference vegetation index (NDVI) (panels **a**–**c**) and green ratio vegetation index (GVI) (panels **d**–**f**) measurements of three cultivars of cucumber (*Cucumis sativus* L. ‘Strategos’, ‘Pradera’ and ‘Mitre’) under three N treatments (N1, N2 and N3). Values are means ± SE.

There were differences between cultivars in NDVI only in the N1 treatment (Table S4). In N2 and N3 treatments, there were no significant differences between the three cultivars (Figure 4b,c). Similar results were found for RVI (Figure S2a–c). In the N1 treatment, ‘Strategos’ and ‘Pradera’ had statistically comparable NDVI values but ‘Strategos’ was significantly different to ‘Mitre’, being, in two measurements date, superior than ‘Mitre’ and in the other two, lower than ‘Mitre’ (Figure 4a). Overall, the average differences in NDVI and GVI values between ‘Strategos’ and ‘Pradera’ with ‘Mitre’ in the N1 treatment were 0.003 ± 0.001 and 0.13 ± 0.014, respectively; expressed as percentage, these average differences were 0.43% and 4.3%, respectively.

For GVI, in N2 treatment during most of the crop, there were statistical differences between ‘Strategos’ and the other two cultivars, with ‘Strategos’ having the lowest values. In the N3 treatment, there were significant differences after 50 DAT, when ‘Pradera’ and ‘Mitre’ had statistically higher GVI values than ‘Strategos’ (Figure 4d–f). There were inconsistent differences between cultivars for GNDVI (Figure S2d–f).

### 3.5. Relationships between Optical Sensor Measurements and Leaf N Content

**Figure 5:**
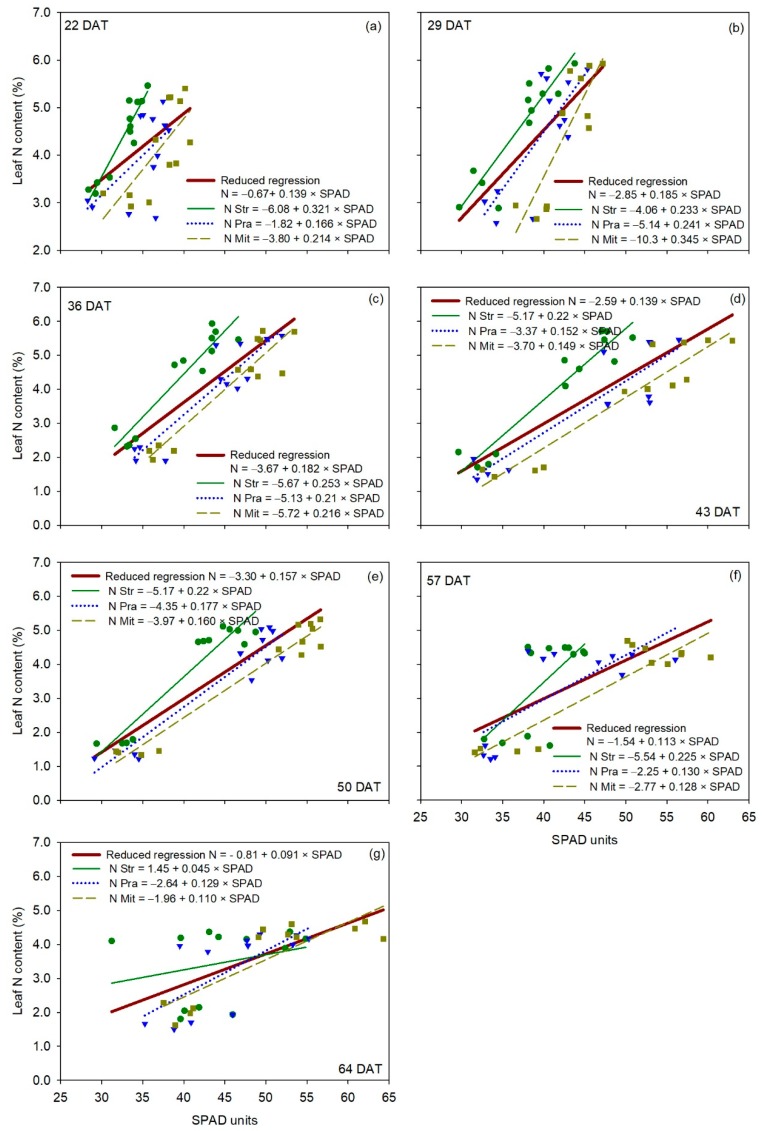
Linear regression between SPAD measurements (independent variable) and leaf N content (dependent variable) for each cultivar of cucumber (*Cucumis sativus* L.), for each measurement date (panels **a**–**g**). The reduced regression is a regression with data of all three cultivars together. DAT is days after transplanting. Str, ‘Strategos’, Pra, ‘Pradera’, Mit, ‘Mitre’.

**Figure 6:**
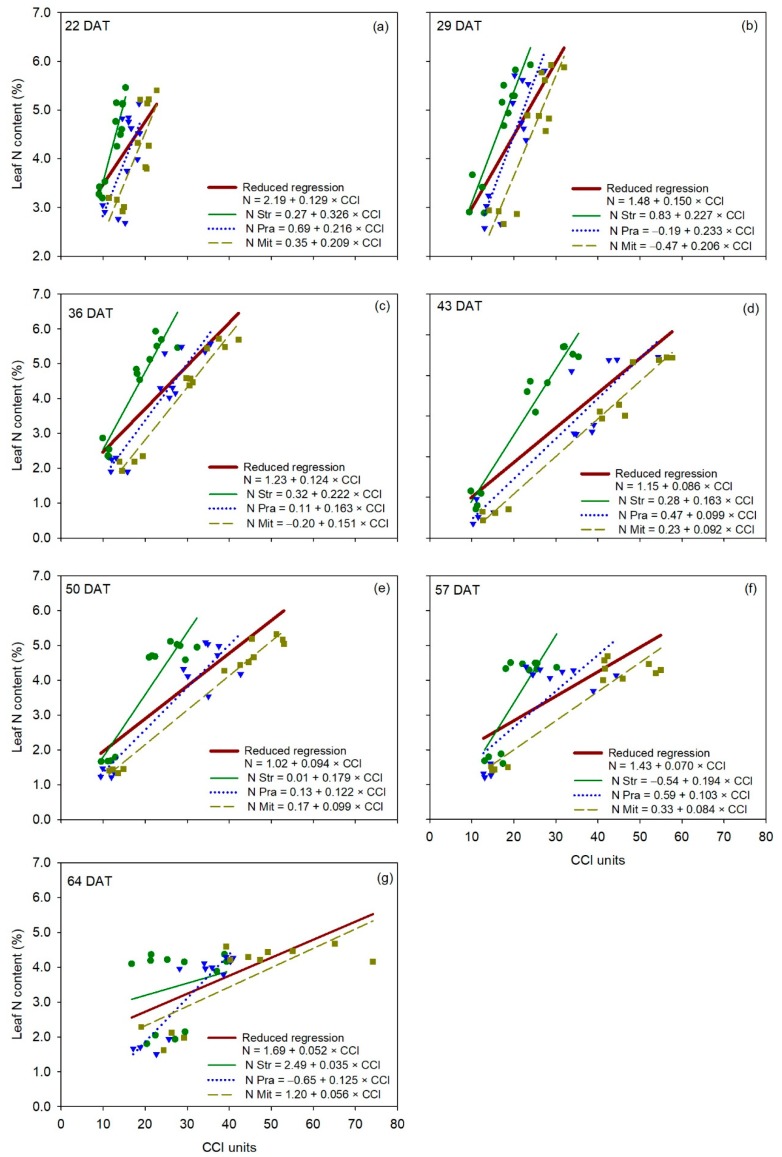
Linear regression between CCI measurements (independent variable) and leaf N content (dependent variable) for each cultivar of cucumber (*Cucumis sativus* L.), for each measurement date (panels **a**–**g**). The reduced regression is a regression with data of all three cultivars together. DAT is days after transplanting. Str, ‘Strategos’, Pra, ‘Pradera’, Mit, ‘Mitre’.

**Figure 7:**
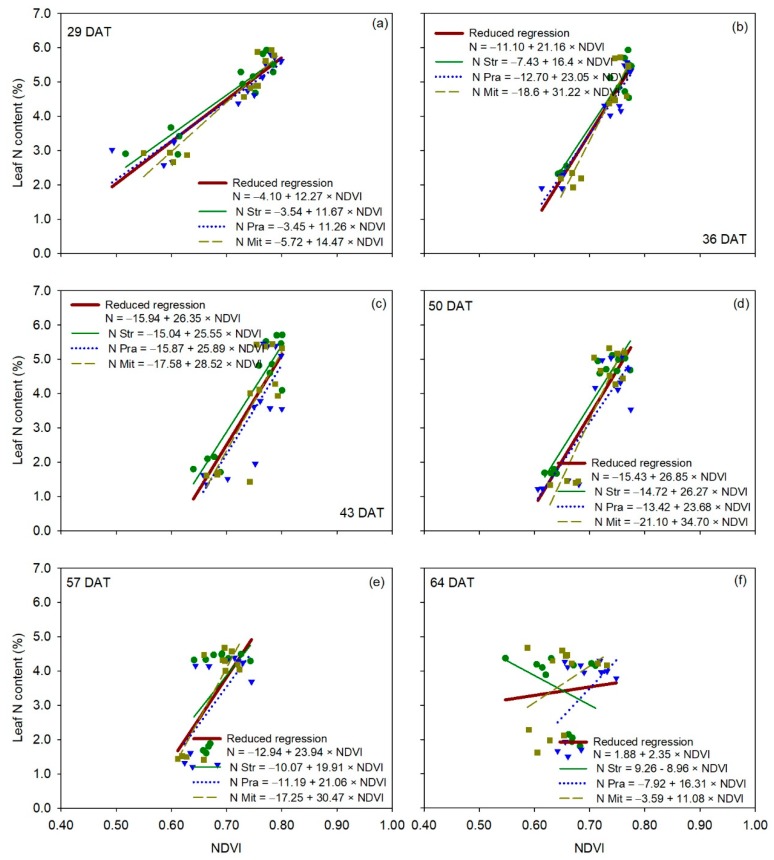
Linear regression between NDVI measurements (independent variable) and leaf N content (dependent variable) for each cultivar of cucumber (*Cucumis sativus* L.), for each measurement date (panels **a**–**f**). The reduced regression is a regression with data of all three cultivars together. DAT is days after transplanting. Str, ‘Strategos’, Pra, ‘Pradera’, Mit, ‘Mitre’

**Figure 8:**
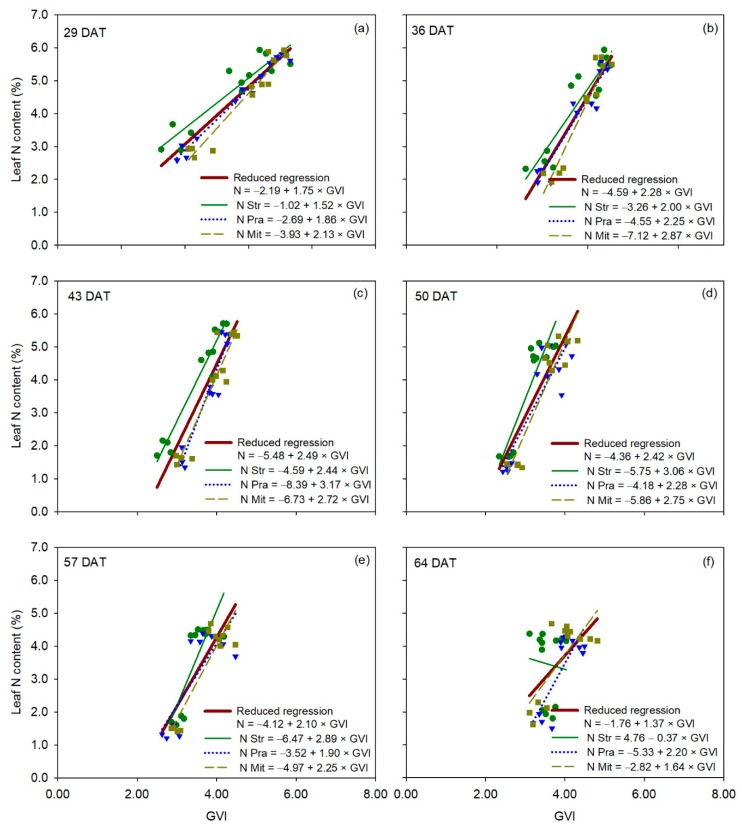
Linear regression between GVI measurements (independent variable) and leaf N content (dependent variable) for each cultivar of cucumber (*Cucumis sativus* L.), for each measurement date (panels **a**–**f**). The reduced regression is a regression with data of all three cultivars together. DAT is days after transplanting. Str, ‘Strategos’, Pra, ‘Pradera’, Mit, ‘Mitre’.

Most of the linear regressions between leaf N content and optical sensor measurements (from chlorophyll meters and the canopy reflectance sensor), for individual measurement dates, were significant for the three cultivars (Figure 5, Figure 6, Figure 7 and Figure 8). On most measurement dates, R^2^ values of the linear regressions were strong or very strong (R^2^ of 0.80–0.98; Table S5). For the SPAD-502, the average R^2^ values of linear regressions, from all measurement dates, were 0.81 ± 0.07, 0.65 ± 0.08 and 0.79 ± 0.06 for ‘Strategos’, ‘Pradera’ and ‘Mitre’, respectively. For CCI, the respective average R^2^ values were 0.83 ± 0.05, 0.74 ± 0.06 and 0.84 ± 0.06. For NDVI, they were 0.85 ± 0.04, 0.72 ± 0.08 and 0.78 ± 0.05, and for GVI were 0.83 ± 0.04, 0.82 ± 0.06 and 0.83 ± 0.05.

For the SPAD-502 (Figure 5), the F-statistic analysis showed that each of the three cultivars had statistically the same linear regression as the reduced regression at 36, 57 and 64 DAT (Table 2), indicating no cultivar effect on the relationship between leaf N content and SPAD measurements in three out of seven measurement dates. ‘Strategos’ had statistically different regressions than the reduced regression at 22, 29 and 43 DAT, and ‘Mitre’ had statistically different regression than the reduced regression at 50 DAT, indicating a significant cultivar effect on the relationship between leaf N content and SPAD measurements in four out of seven measurement dates (Table 2). By contrast, the regression of ‘Pradera’ was statistically similar to the reduced regression for all measurement dates. 

The F-statistic analysis showed that the reduced regression between leaf N content and measurements of the MC-100 was statistically comparable to the individual regressions for each cultivar at 64 DAT, indicating no cultivar effect on the relationship between leaf N content and MC-100 measurements in one of seven measurement dates (Figure 6). ‘Strategos’ and ‘Mitre’ had significantly different regressions to the reduced regression at 22, 29 and 43 DAT, and at 36, 43, 50 and 57 DAT, respectively (Table 2). The regression of ‘Pradera’ was statistically similar to the reduced regression on all measurement dates (Table 2).

For canopy reflectance vegetation indices, the relationship between leaf N content and NDVI was statistically comparable between the reduced regression for all three cultivars and each of the individual regressions for each of the three cultivars for all measurement dates (Table 2), indicating no significant cultivar effect on the relationship between leaf N content and NDVI (Figure 7). Very similar behavior to that of NDVI occurred with RVI and GNDVI (Table S6 and Figures S3 and S4). Overall, the results of the F-statistic analysis for GVI were very similar to those of NDVI (Figure 8), without significant differences between the reduced regression for all three cultivars together and the individual regression for each cultivar, on five out of six measurement dates (Table 2). Regressions for ‘Strategos’ and ‘Pradera’ were statistically different to the reduced regression on 43 and 29 DAT, respectively (Table 2).

## 4. Discussion

There were differences between cultivars, for equivalent N treatments, of measurements made with the SPAD-502 and MC-100 chlorophyll meters, when the N supply was sufficient and excessive (N2 and N3 treatments), but not when the N supply was deficient (N1 treatment). There are previous reports of cultivar notably affecting SPAD measurements in wheat [30] and rice [32]. For the vegetation indices measured with the Crop Circle ACS-470 reflectance sensor, there were no consistent significant differences between cultivars. 

The general similarities, for the three cultivars, in the slopes of the linear relationships between sensor measurements and leaf N content, for the three optical sensors, indicated that the sensitivity of the two chlorophyll meters and the canopy reflectance sensor was not affected by cultivar. However, there were significant differences in relationships between the reduced regression for all three cultivars considered together and the regressions for individual cultivars, particularly for chlorophyll meters. This indicated a significant cultivar effect on the relationship between leaf N content and optical sensor measurements. It suggested that a unique regression equation to estimate leaf N content from sensor measurement could not be used for each of the three cucumber cultivars examined in the present work. These results are subsequently discussed more fully. 

### 4.1. Assessment of Cultivar Effects on Optical Sensor Measurements

With both chlorophyll meters, there were consistent differences in measurements between the three cultivars, mainly between ‘Mitre’ and ‘Strategos’, with ‘Pradera’ being intermediate. These differences between cultivars were most apparent in the sufficient and excessive N treatments (N2 and N3). These results are consistent with previous work with other species where cultivar effects on SPAD measurements were more pronounced at higher N supply, in rice [44], potato [45] and tomato [33]. There are no previous reports evaluating cultivar effects on measurements made with the MC-100 chlorophyll meter. 

In the present work, the use of two different chlorophyll meters enabled the relative effect of cultivar on the two sensors to be compared. The differences in measurement between cultivars were appreciably larger with the MC-100 compared to the SPAD-502. For example, in the N3 treatment, the average relative difference between ‘Mitre’ and ‘Strategos’ was 42% with the MC-100 meter, and 17% with the SPAD-502 meter. The relative differences between ’Mitre’ and ‘Strategos’ cultivars were slightly lower than the relative differences in measurements between the N1 and N2 treatments and appreciably larger than those between the N2 and N3 treatments. These results contradict the observation of Hoel [31] that the soil N availability affected chlorophyll meter readings more than cultivar, growth stage and other nutrients in wheat.

The cultivar effect observed with chlorophyll meters, in the current study, has implications for the use of absolute sufficiency values, of chlorophyll meter measurements, as indicators of optimal crop N status. Sufficiency values (also known as reference or threshold values) being those that distinguish between deficiency (below the value) and sufficiency (above the value) [8]. Monostori et al. [30] reported for wheat that SPAD values should be calibrated for each cultivar to obtain more accurate N diagnosis and yield prediction. The present work and previous research [46,47] suggest that in order to use absolute sufficiency values, regardless of the cultivar, that procedures to normalize absolute chlorophyll meter measurements should be developed.

The relative differences between cultivars in vegetation indices measured with Crop Circle ACS-470 sensor were much smaller and less consistent than occurred with chlorophyll meter measurement. For NDVI, statistical differences between cultivars were detected in N1, but not in N2 and N3 treatments, which was the opposite to what was observed with chlorophyll meters. Cultivar differences in NDVI were reported by Sultana et al. [17] who observed significant differences in NDVI between wheat cultivars under four different nitrogen levels. Similar results for geranium (*Pelargonium × hortorum*) were reported by Wang et al. [48]. The lack of consistent differences in the N1 treatment, in the present work, may be due to the limited vegetative growth of this treatment, the lack of continuity of vegetative cover may have influenced canopy reflectance. Padilla et al. [5], Wang et al. [48] and Johansen and Tømmervik [49], reported that NDVI is susceptible to measurement error caused by background reflectance when the canopy is not sufficiently closed. Comparing the LAI between the different cultivars, ‘Strategos’ had the highest LAI values, but this did not influence vegetation indices; the values of vegetation indices were generally comparable, in statistical terms, between cultivars. This suggests that not only the quantity of leaves has an influence on reflectance measurements but also other plant characteristics such as the angle position of leaves [10].

A factor that affected canopy reflectance measurements in the final stages of the crops, in the present study (after 50 DAT), was foliar damage due to fungal infection of powdery mildew (*Pseudoperonospora cubensis*), which marked an appreciable portion of the leaves with yellow spots, mostly in the N2 and N3 treatments. This foliar damage could have influenced the decrease in reflectance indices in the three cultivars, which was most apparent in the cultivar ‘Strategos’. This was consistent with the relatively large increase in reflectance of the red and green bands towards the end of the crop. Similar results were found in soybean, where a decline in NDVI was strongly related to foliar damage [50].

Considering the entire data set, of canopy reflectance measurement, in the current study, the vegetation indices using the green wavelength (GNDVI and GVI) were more sensitive than the red indices (NDVI and RVI) for detecting cultivar differences. This is in agreement with Padilla et al. [28], where the GNDVI and GVI indices were the most sensitive vegetation indices for estimating both crop nitrogen nutrition index (NNI) and yield in cucumber. With processing tomato, green vegetation indices were also more sensitive than red vegetation indices for estimating leaf N content [25]. Loss of sensitivity of red vegetation indices related to saturation of reflectance in the red region at high leaf area index (LAI) values has been reported in different field crop species such as wheat, soybean and maize [51]. However, in the present study, differential saturation of the red and green bands during the crop was not observed (Figure 4a–f).

### 4.2. Relationships Between Optical Sensor Measurements and Leaf N Content

The strong relationships between chlorophyll meter measurements (both SPAD-502 and MC-100) and leaf N content indicated that these measurements were good indicators of leaf N content, for the cultivars examined. These results are consistent with previous research in which chlorophyll meter measurements were strongly related to leaf N content [5,52,53]. Comparing chlorophyll meter measurements with the parameters measured with the colorimeter, the results were apparently contradictory. ‘Strategos’ was the cultivar with lowest chlorophyll meter measurements while having the highest luminance and chromatic coordinates. It may be that higher luminance measured with the colorimeter in ‘Strategos’ is indicative of lower light absorption and higher light transmittance and reflectance, as indicated by the lower chlorophyll meter measurements and vegetation indices values of this cultivar. 

Similarly, the generally strong relationships, between NDVI, RVI, GNDVI and GVI, and leaf N content, indicated that these vegetation indices are effective indicators of leaf N content in cucumber, for the cultivars examined. Padilla et al. [28] reported that these vegetation indices were good estimators of crop N status in cucumber. Previous studies in tomato and geranium have reported strong relationship between vegetation indices such as NDVI and GNDVI with leaf N content [25,48].

Significant differences were found between the reduced regression for all three cultivars considered together and the individual regressions for ‘Strategos’ and ‘Mitre’ considered separately, with the SPAD-502 and MC-100, for most measurement dates. This indicated a significant cultivar effect on the relationships between chlorophyll meter measurement and leaf N. Consequently, it appears that it is not feasible to use a unique equation for the three cultivars to estimate leaf N content from chlorophyll meter measurements. These results imply that procedures to normalize differences between cultivars should be developed in order to use absolute sufficiency values developed for a given species. 

For canopy reflectance, the lack of significant differences between the reduced regression for all three cultivars together and the regressions for each of the three cultivars separately, for most measurement dates, indicated that there was not a significant cultivar effect on the relationship between leaf N content and vegetation indices in cucumber. This suggested that a single regression equation could be used to estimate leaf N content, for the three cultivars, for measurements of NDVI, GNDVI, RVI and GVI. 

## 5. Conclusions

Cultivar had an effect on SPAD-502 and MC-100 chlorophyll meter measurements when the N supply was adequate and excessive. For the red band based vegetation indices (NDVI and RVI) measured with the Crop Circle ACS470 sensor, there was no effect of cultivar, regardless of N applied. For the green band based vegetation indices (GNDVI and GVI), there was a cultivar effect, mainly with ‘Strategos’, which indicated it is not possible to use a unique sufficiency value for the three cultivars. Cultivar had a significant effect on the relationship between leaf N content and chlorophyll meter measurements, but not on the relationships between leaf N content and canopy reflectance vegetation indices. The lack of a consistent effect of cultivar, on the relationship with leaf N content, suggests that a unique equation to estimate leaf N content from vegetation indices can be applied to all three cultivars. This unique equation, however, may not be applied for chlorophyll meter measurements because of the significant cultivar effect detected in the present study.

## Figures and Tables

**Table 1:** Averages of the three N treatments of leaf area index (LAI), crop height, luminance (Y), coordinate x and coordinate y for each cultivar of cucumber (*Cucumis sativus* L.) crop grown in 2018. Values are means ± standard error. There were twelve measurements of each parameter for each cultivar, three for each N treatment. Different lower-case letters (a–c) show significant differences between cultivars.

Cultivar	LAI	Crop Height (m)	Luminance (Y)	Coordinate x	Coordinate y
‘Strategos’	5.68 ± 0.69 ^a^	1.75 ± 0.11 ^a^	10.47 ± 0.72 ^a^	0.331 ± 0.003 ^a^	0.401 ± 0.007 ^a^
‘Pradera’	5.20 ± 0.74 ^b^	1.71 ± 0.12 ^a^	9.57 ± 0.84 ^b^	0.330 ± 0.003 ^a,b^	0.396 ± 0.008 ^b^
‘Mitre’	4.98 ± 0.70 ^b^	1.72 ± 0.11 ^a^	8.94 ± 0.70 ^c^	0.328 ± 0.003 ^b^	0.390 ± 0.007 ^c^

**Table 2:** *p*-values of the F-statistic analysis comparing the relationship between leaf N content and optical sensor measurements between the reduced regression for all three cultivars together and the regression of each cultivar of cucumber (*Cucumis sativus* L.) separately. Numbers in bold show significant differences (*p* < 0.05) between the reduced regression and the cultivar regression.

DAT	SPAD			CCI			NDVI			GVI		
	‘Strategos’	‘Pradera’	‘Mitre’	‘Strategos’	‘Pradera’	‘Mitre’	‘Strategos’	‘Pradera’	‘Mitre’	‘Strategos’	‘Pradera’	‘Mitre’
22	**0.005**	0.350	0.262	**0.009**	0.281	0.177						
29	**0.046**	0.281	0.215	**0.046**	0.201	0.131	0.262	0.409	0.281	0.350	**0.019**	0.166
36	0.070	0.139	0.098	0.057	0.083	**0.001**	0.245	0.262	0.377	0.350	0.189	0.229
43	**0.048**	0.201	0.078	**0.015**	0.103	**0.001**	0.147	0.409	0.444	**0.041**	0.087	0.189
50	0.116	0.131	**0.034**	0.123	0.123	**<0.001**	0.098	0.409	0.350	0.201	0.281	0.215
57	0.377	0.324	0.078	0.229	0.262	**0.032**	0.444	0.377	0.177	0.324	0.377	0.166
64	0.484	0.302	0.147	0.484	0.054	0.229	0.281	0.281	0.324	0.444	0.166	0.215

## Supplementary Materials

The following are available online at https://www.mdpi.com/1424-8220/20/2/509/s1, Figure S1: Chromatic differences (x coordinate and y coordinate of CIE 1931 color space) between (a) cultivars, when pooling over the three N treatments, and between (b) N treatments, when pooling over the three cultivars, of a cucumber (*Cucumis sativus* L.) crop under three N treatments. Values are means ± SE. Pooling was possible because of not significant Cultivar x Nitrogen interaction for x and y coordinates (see Table 1), Figure S2: Temporal dynamics of RVI and GNDVI measurements of three cultivars of cucumber (*Cucumis sativus* L. ‘Strategos’, ‘Pradera’ and ‘Mitre’) under three N treatments (N1, N2 and N3). Values are means ± SE, Figure S3: Linear regression between RVI measurements (independent variable) and leaf N content (dependent variable) for each cultivar of cucumber (*Cucumis sativus* L.), for each measurements date. The reduced regression is a regression with data of all three cultivars together. DAT is days after transplanting. Str, ‘Strategos’, Pra, ‘Pradera’, Mit, ‘Mitre’, Figure S4: Linear regression between GNDVI measurements (independent variable) and leaf N content (dependent variable) for each cultivar of cucumber (*Cucumis sativus* L.), for each measurements date. The reduced regression is a regression with data of all three cultivars together. DAT is days after transplanting. Str, ‘Strategos’, Pra, ‘Pradera’, Mit, ‘Mitre’, Table S1: Analysis of variance testing the effect of cultivar and nitrogen on crop leaf area index (LAI), crop height, luminance (Y) and chromatic coordinates xy of CIE 1931 color space, of a cucumber (*Cucumis sativus* L.) crop, Table S2: Analysis of variance testing the effect of cultivar, nitrogen and time, on leaf N content of cucumber (*Cucumis sativus* L.) crop, Table S3: Analysis of variance testing the effect of cultivar, nitrogen and time, on SPAD and CCI measurements of cucumber (*Cucumis sativus* L.) crop, Table S4: Analysis of variance testing the effect of cultivar, nitrogen and time, on NDVI, GNDVI, RVI and GVI measurements of cucumber (*Cucumis sativus* L.) crop, Table S5: Coefficients of determination (R^2^) and standard error of the estimate (SEE) of linear regression between each optical sensor measurements (independent variable) and leaf N content (dependent variable) for each cultivar of cucumber (*Cucumis sativus* L.). DAT is days after transplanting. Symbols close to R^2^ values show significance of linear regression (ns, not significant at *p* ≥ 0.05; *, *p* < 0.05; **, *p* < 0.01; ***, *p* < 0.001), Table S6: P-values of the F-statistic analysis comparing the relationship between leaf N content and optical sensor measurements between the reduced regression for all three cultivars together and the regression of each cultivar of cucumber (*Cucumis sativus* L.) separately.

## References

[1] Ju X.T., Kou C.L., Zhang F.S., Christie P. (2006). Nitrogen balance and groundwater nitrate contamination: Comparison among three intensive cropping systems on the North China Plain. Environ. Pollut..

[2] Thompson R.B., Martínez-Gaitan C., Gallardo M., Giménez C., Fernández M.D. (2007). Identification of irrigation and N management practices that contribute to nitrate leaching loss from an intensive vegetable production system by use of a comprehensive survey. Agric. Water Manag..

[3] Zotarelli L., Dukes M.D., Scholberg J.M.S., Muñoz-Carpena R., Icerman J. (2009). Tomato nitrogen accumulation and fertilizer use efficiency on a sandy soil, as affected by nitrogen rate and irrigation scheduling. Agric. Water Manag..

[4] Meisinger J.J., Schepers J.S., Raun W.R. (2008). Crop Nitrogen Requirement and Fertilization. Am. Soc. Agron. Crop Sci. Soc. Am. Soil Sci. Soc. Am..

[5] Padilla F.M., Gallardo M., Peña-Fleitas M.T., de Souza R., Thompson R.B. (2018). Proximal Optical Sensors for Nitrogen Management of Vegetable Crops: A Review. Sensors.

[6] Pulido-Bosch A., Bensi S., Molina L., Vallejos A., Calaforra J.M., Pulido-Leboeuf P. (2000). Nitrates as indicators of aquifer interconnection. Application to the Campo de Dalias (SE - Spain). Environ. Geol..

[7] Cui M., Sun X., Hu C., Di H.J., Tan Q., Zhao C. (2011). Effective mitigation of nitrate leaching and nitrous oxide emissions in intensive vegetable production systems using a nitrification inhibitor, dicyandiamide. J. Soils Sediments.

[8] Thompson R.B., Tremblay N., Fink M., Gallardo M., Padilla F.M., Tei F., Nicola S., Benincasa P. (2017). Tools and strategies for sustainable nitrogen fertilisation of vegetable crops. Advances in Research on Fertilization Management in Vegetable Crops.

[9] Schröder J.J., Neeteson J.J., Oenema O., Struik P.C. (2000). Does the crop or the soil indicate how to save nitrogen in maize production? Reviewing the state of the art. Field Crops Res..

[10] Fox R.H., Walthall C.L., Schepers J.S., Raun W.R. (2008). Crop monitoring technologies to assess nitrogen status. Nitrogen in Agricultural Systems, Agronomy Monograph No. 49.

[11] Samborski S.M., Tremblay N., Fallon E. (2009). Strategies to make use of plant sensors-based diagnostic information for nitrogen recommendations. Agron. J..

[12] Schepers J.S., Blackmer T.M., Wilhelm W.W., Resende M. (1996). Transmittance and reflectance measurements of corn leaves from plants with different nitrogen and water supply. J. Plant Physiol..

[13] Gianquinto G., Goffart J.P., Olivier M., Guarda G., Colauzzi M., Dalla Costa L., Delle Vedove G., Vos J., Mackerron D.K.L. (2004). The use of hand-held chlorophyll meters as a tool to assess the nitrogen status and to guide nitrogen fertilization of potato crop. Potato Res..

[14] Ollinger S. (2011). V Sources of variability in canopy reflectance and the convergent properties of plants. New Phytol..

[15] Bannari A., Morin D., Bonn F., Huete A.R. (1995). A review of vegetation indices. Remote Sens. Rev..

[16] Hatfield J.L., Prueger J.H. (2010). Value of using different vegetative indices to quantify agricultural crop characteristics at different growth stages under varying management practices. Remote Sens..

[17] Sultana S.R., Ali A., Ahmad A., Mubeen M., Zia-Ul-Haq M., Ahmad S., Ercisli S., Jaafar H.Z.E. (2014). Normalized difference vegetation index as a tool for wheat yield estimation: A case study from Faisalabad, Pakistan. Sci. World J..

[18] Wakiyama Y. (2016). The relationship between SPAD values and leaf blade chlorophyll content throughout the rice development cycle. Jpn. Agric. Res. Q..

[19] Blackmer T.M., Schepers J.S. (1995). Use of a chlorophyll meter to monitor nitrogen status and schedule fertigation for corn. J. Prod. Agric..

[20] Ziadi N., Bélanger G., Claessens A., Lefebvre L., Tremblay N., Cambouris A.N., Nolin M.C., Parent L.E. (2010). Plant-based diagnostic tools for evaluating wheat nitrogen status. Crop Sci..

[21] Debaeke P., Rouet P., Justes E. (2006). Relationship between the normalized SPAD index and the nitrogen nutrition index: Application to durum wheat. J. Plant Nutr..

[22] Mistele B., Schmidhalter U. (2008). Estimating the nitrogen nutrition index using spectral canopy reflectance measurements. Eur. J. Agron..

[23] Olivier M., Goffart J.P., Ledent J.F. (2006). Threshold value for chlorophyll meter as decision tool for nitrogen management of potato. Agron. J..

[24] Gianquinto G., Sambo P., Bona S. (2003). The use of SPAD-502 chlorophyll meter for dynamically optimising the nitrogen supply in potato crop: A methodological approach. Acta Hortic..

[25] Gianquinto G., Orsini F., Fecondini M., Mezzetti M., Sambo P., Bona S. (2011). A methodological approach for defining spectral indices for assessing tomato nitrogen status and yield. Eur. J. Agron..

[26] Güler S., Büyük G. (2007). Relationships among chlorophyll-meter reading value, leaf N and yield of cucumber and tomatoes. Acta Hortic..

[27] Padilla F.M., Peña-Fleitas M.T., Gallardo M., Thompson R.B. (2015). Threshold values of canopy reflectance indices and chlorophyll meter readings for optimal nitrogen nutrition of tomato. Ann. Appl. Biol..

[28] Padilla F.M., Peña-Fleitas M.T., Gallardo M., Thompson R.B. (2017). Determination of sufficiency values of canopy reflectance vegetation indices for maximum growth and yield of cucumber. Eur. J. Agron..

[29] Padilla F.M., Peña-Fleitas M.T., Gallardo M., Thompson R.B. (2014). Evaluation of optical sensor measurements of canopy reflectance and of leaf flavonols and chlorophyll contents to assess crop nitrogen status of muskmelon. Eur. J. Agron..

[30] Monostori I., Árendás T., Hoffman B., Galiba G., Gierczik K., Szira F., Vágújfalvi A. (2016). Relationship between SPAD value and grain yield can be affected by cultivar, environment and soil nitrogen content in wheat. Euphytica.

[31] Hoel B.O. (2003). Chlorophyll meter readings in winter wheat: Cultivar differences and prediction of grain protein content. Acta Agric. Scand. Sect. B Soil Plant Sci..

[32] Peng S., Garcia F.V., Laza R.C., Cassman K.G. (1993). Adjustment for specific leaf weight improves chlorophyll meter’s estimate of rice leaf nitrogen concentration. Agron. J..

[33] Sandoval-Villa M., Guertal E.A., Wood C.W. (2000). Tomato leaf chlorophyll meter readings as affected by variety, nitrogen form, and nighttime nutrient solution strength. J. Plant Nutr..

[34] Samborski S.M., Gozdowski D., Walsh O.S., Lamb D.W., Stępień M., Gacek E.S., Drzazga T. (2015). Winter wheat genotype effect on canopy reflectance: Implications for using NDVI for in-season nitrogen topdressing recommendations. Agron. J..

[35] Padilla F.M., Peña-Fleitas M.T., Gallardo M., Thompson R.B. (2016). Proximal optical sensing of cucumber crop N status using chlorophyll fluorescence indices. Eur. J. Agron..

[36] Padilla F.M., Thompson R.B., Peña-Fleitas M.T., Gallardo M. (2018). Reference values for phenological phases of chlorophyll meter readings and reflectance indices for optimal N nutrition of fertigated tomato. Acta Hortic..

[37] Solari F., Shanahan J., Ferguson R., Schepers J., Gitelson A. (2008). Active sensor reflectance measurements of corn nitrogen status and yield potential. Agron. J..

[38] Sellers P.J. (1985). Canopy reflectance, photosynthesis and transpiration. Int. J. Remote Sens..

[39] Ma B.L., Morrison M.J., Dwyer L.M. (1996). Canopy light reflectance and field greenness to assess nitrogen fertilization and yield of maize. Agron. J..

[40] Birth G.S., McVey G.R. (1968). Measuring the color of growing turf with a reflectance spectrophotometer. Agron. J..

[41] Fitzgerald G.J. (2010). Characterizing vegetation indices derived from active and passive sensors. Int. J. Remote Sens..

[42] Hatfield J.L., Gitelson A.A., Schepers J.S., Walthall C.L. (2008). Application of spectral remote sensing for agronomic decisions. Agron. J..

[43] ArchMiller A.A., Samuelson L.J. (2016). Intra-annual variation of soil respiration across four heterogeneous longleaf pine forests in the southeastern United States. For. Ecol. Manage..

[44] Yuan Z., Ata-Ul-Karim S.T., Cao Q., Lu Z., Cao W., Zhu Y., Liu X. (2016). Indicators for diagnosing nitrogen status of rice based on chlorophyll meter readings. Field Crops Res..

[45] Minotti P.L., Halseth D.E., Sieczka J.B. (1994). Field chlorophyll measurements to assess the nitrogen status of potato varieties. HortScience.

[46] Zhao B., Liu Z.Z., Ata-Ul-Karim S.T., Xiao J., Liu Z.Z., Qi A., Ning D., Nan J., Duan A. (2016). Rapid and nondestructive estimation of the nitrogen nutrition index in winter barley using chlorophyll measurements. Field Crops Res..

[47] Liu X., Ferguson R.B., Zheng H., Cao Q., Tian Y., Cao W., Zhu Y. (2017). Using an active-optical sensor to develop an optimal NDVI dynamic model for high-yield rice production (Yangtze, China). Sensors.

[48] Wang Y.W., Mao P.S., Dunn B.L., Arnall D.B. (2012). Use of an active canopy sensor and SPAD chlorophyll meter to quantify geranium nitrogen status. HortScience.

[49] Johansen B., Tømmervik H. (2014). The relationship between phytomass, NDVI and vegetationcommunities on Svalbard. Int. J. Appl. Earth Obs. Geoinf..

[50] Hikishima M., Giovanetti Canteri M., Godoy C.V., Koga L.J., da Silva A.J. (2011). Quantificação de danos e relações entre severidade, medidas de refletância e produtividade no patossistema ferrugem asiática da soja. Trop. Plant Pathol..

[51] Gitelson A. (2004). Wide Dynamic Range Vegetation Index for Remote Quantification of Biophysical Characteristics of Vegetation. J. Plant Physiol..

[52] Esfahani M., Abbasi H.R.A., Rabiei B., Kavousi M. (2008). Improvement of nitrogen management in rice paddy fields using chlorophyll meter (SPAD). Paddy Water Environ..

[53] Castelli F., Contillo R. (2009). Using a Chlorophyll Meter to Evaluate the Nitrogen Leaf Content in Flue-Cured Tobacco (*Nicotiana tabacum* L.). Ital. J. Agron..

