# Supplementary material for: Effect of Cultivar on Chlorophyll Meter and Canopy Reflectance Measurements in Cucumber
Source: Sensors (Basel). 2020 Jan 16;20(2):509. doi: 10.3390/s20020509 (PMC7014412; doi:10.3390/s20020509)
Supplement: Supplementary file 1 [file sensors-20-00509-s001.pdf]

Supplementary material

Table S1: Analysis of variance testing the effect of cultivar and nitrogen on crop leaf area index (LAI), crop height, luminance (Y) and chromatic coordinates xy of CIE 1931 color space, of a cucumber (*Cucumis sativus* L.) crop.

		**LAI**		**Crop height**		**Luminance (Y)**		**Coordinate x**		**Coordinate y**	
**Effect**	**df**	F	P	**F**	**P**	**F**	**P**	**F**	**P**	**F**	**P**
Block	3	1.13	0.356	2.48	0.085	1.60	0.215	1.02	0.403	0.68	0.573
Cultivar (C)	2	6.32	0.006	0.65	0.532	22.32	<0.001	7.44	0.003	11.38	<0.001
Nitrogen (N)	2	373.40	<0.001	272.85	<0.001	347.55	<0.001	504.19	<0.001	372.69	<0.001
C x N	4	1.10	0.379	1.61	0.204	1.98	0.130	2.21	0.10	2.26	0.092
Error	24										

Table S2: Analysis of variance testing the effect of cultivar, nitrogen and time, on leaf N content of cucumber (*Cucumis sativus* L.) crop.

		**F**	**P**
Block	3	3.46	0.032
Cultivar (C)	2	29.40	<0.001
Nitrogen (N)	2	2441.02	<0.001
C x N	4	3.73	0.017
Error	24		
Time (T)	6	257.75	<0.001
T x C	12	3.71	<0.001
T x N	12	73.01	<0.001
T x C x N	24	1.88	<0.013
Error	144		

Table S3: Analysis of variance testing the effect of cultivar, nitrogen and time, on SPAD and CCI measurements of cucumber (*Cucumis sativus* L.) crop.

		SPAD		CCI	
**Effect**	**df**	**F**	**P**	**F**	**P**
Block	3	14.12	<0.001	8.55	<0.001
Cultivar (C)	2	72.75	<0.001	110.37	<0.001
Nitrogen (N)	2	310.88	<0.001	277.20	<0.001
C x N	4	7.34	<0.001	15.58	<0.001
Error	24				
Time (T)	6	111.07	<0.001	134.15	<0.001
T x C	12	1.62	0.091	5.72	<0.001
T x N	12	23.47	<0.001	30.94	<0.001
T x C x N	24	3.01	<0.001	3.05	<0.001
Error	144				

Table S4: Analysis of variance testing the effect of cultivar, nitrogen and time, on NDVI, GNDVI, RVI and GVI measurements of cucumber (*Cucumis sativus* L.) crop.

		NDVI		GNDVI		RVI		GVI	
	**df**	**F**	**P**	**F**	**P**	**F**	**P**	**F**	**P**
Block	3	28.52	<0.001	5.36	0.006	30.01	<0.001	6.28	0.003
Cultivar (C)	2	3.30	0.054	10.46	<0.001	4.71	0.019	18.25	<0.001
Nitrogen (N)	2	469.37	<0.001	442.93	<0.001	375.34	<0.001	331.41	<0.001
C x N	4	1.52	0.228	1.98	0.129	0.84	0.516	1.43	0.256
Error	24								
Time (T)	5	89.51	<0.001	61.28	<0.001	197.22	<0.001	67.18	<0.001
T x C	10	3.08	0.002	2.93	0.003	2.41	<0.012	2.58	0.007
T x N	10	42.44	<0.001	27.92	<0.001	70.81	<0.001	28.47	<0.001
T x C x N	20	2.96	<0.001	3.42	<0.001	4.57	<0.001	4.54	<0.001
Error	120								

Table S5: Results of linear regression analysis between GNDVI measurements (independent variable) and leaf N content (dependent variable) for each cultivar of cucumber (*Cucumis sativus* L.) crop.

Crop	DAT	'Strategos'				'Pradera'				'Mitre'			
		R^2	±SEE	Slope	Intercept	R^2	±SEE	Slope	Intercept	R^2	±SEE	Slope	Intercept
2018	29	0.89***	0.39	15.49a	-3.97A	0.96***	0.25	19.03a	-6.40B	0.92***	0.38	21.33a	-7.73B
	36	0.91***	0.44	21.42a	-7.87A	0.94***	0.36	23.19a	-9.04A	0.90***	0.50	27.43a	-11.60A
	43	0.92***	0.48	24.39a	-9.29A	0.87***	0.60	25.46a	-10.69B	0.90***	0.55	24.36a	-10.05B
	50	0.79***	0.75	24.45a	-8.53AB	0.86***	0.64	19.94a	-6.88A	0.90***	0.57	24.93a	-9.61B
	57	0.73***	0.71	27.68a	-11.36A	0.76***	0.72	19.19a	-7.14B	0.90***	0.46	22.68a	-9.24AB
	64	0.05 ns	1.11	-9.24	8.53	0.42*	0.94	20.88a	-8.88A	0.65**	0.75	21.50a	-8.80A

DAT is days after transplanting. R^2 is coefficient of determination of linear regression and SEE is standard error of the estimation (%N). Symbols close to R^2 values show significance of linear regression (ns, not significant at $p \geq 0.05$; *, $p<0.05$; **, $p<0.01$; ***, $p<0.001$) For each day, different lower-case letters show significant differences in slope of linear regression between cultivars, and different upper-case letters show significant differences in intercept of linear regression between cultivars. Comparison of slope and intercept of linear regressions between cultivars has been done for significant regressions only. Sample size of regressions for each date and cultivar was 12.

Table S6. Results of linear regression analysis between RVI measurements (independent variable) and leaf N content (dependent variable) for each cultivar of cucumber (*Cucumis sativus* L.) crop.

DAT	'Strategos'				'Pradera'				'Mitre'			
	R^2	±SEE	Slope	Intercept	R^2	±SEE	Slope	Intercept	R^2	±SEE	Slope	Intercept
29	0.88***	0.41	0.57a	0.92A	0.94***	0.32	0.58a	0.51B	0.95***	0.31	0.74a	-0.44C
36	0.87***	0.52	0.89a	-1.79A	0.89***	0.51	0.96a	-2.56A	0.84***	0.64	1.20a	-4.08A
43	0.86***	0.64	0.72a	-1.25A	0.61**	1.04	0.82a	-2.74A	0.74***	0.89	0.91a	-3.00A
50	0.81***	0.71	0.93a	-2.04A	0.64**	1.03	0.88a	-2.39A	0.72***	0.95	1.27a	-4.52A
57	0.31 ns	1.15	1.02	-2.25	0.45*	1.08	0.10a	-2.64A	0.70***	0.81	1.60b	-5.64B
64	0.01 ns	1.14	-0.19	4.44	0.38*	0.97	0.87	-1.82	0.27 ns	1.07	0.74	-0.30

DAT is days after transplanting. R^2 is coefficient of determination of linear regression and SEE is standard error of the estimation (%N). Symbols close to R^2 values show significance of linear regression (ns, not significant at $p \geq 0.05$; *, $p < 0.05$; **, $p < 0.01$; ***, $p < 0.001$) For each day, different lower-case letters show significant differences in slope of linear regression between cultivars, and different upper-case letters show significant differences in intercept of linear regression between cultivars. Comparison of slope and intercept of linear regressions between cultivars has been done for significant regressions only. Sample size of regressions for each date and cultivar was 12.

Figures

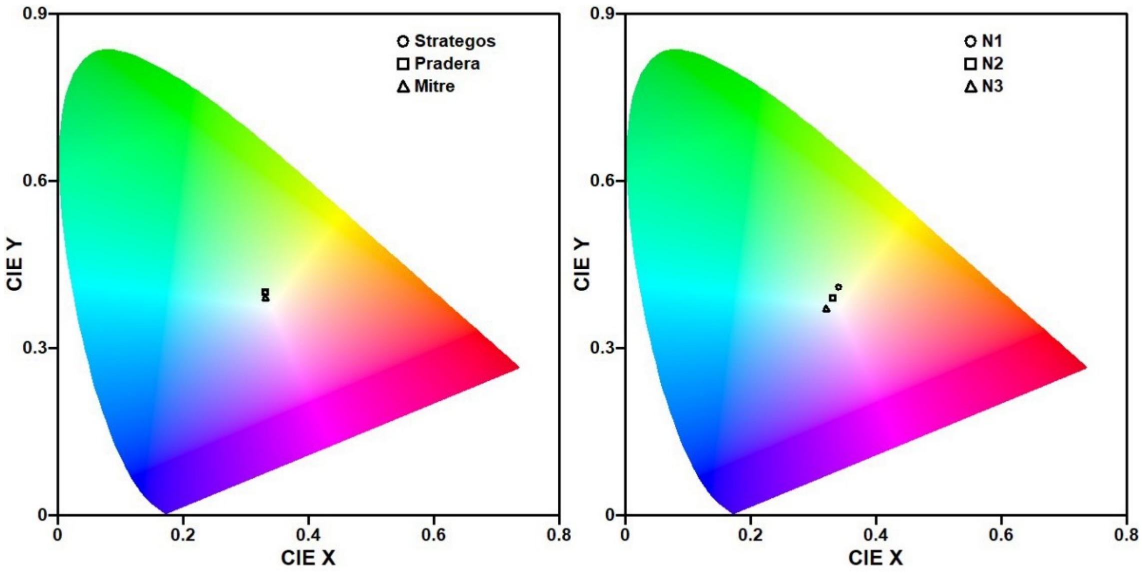

Figure S1. Chromatic differences (x coordinate and y coordinate of CIE 1931 color space) between (a) cultivars, when pooling over the three N treatments, and between (b) N treatments, when pooling over the three cultivars, of a cucumber (*Cucumis sativus* L.) crop under three N treatments. Values are means ± SE. Pooling was possible because of not significant Cultivar x Nitrogen interaction for x and y coordinates (see Table 1).

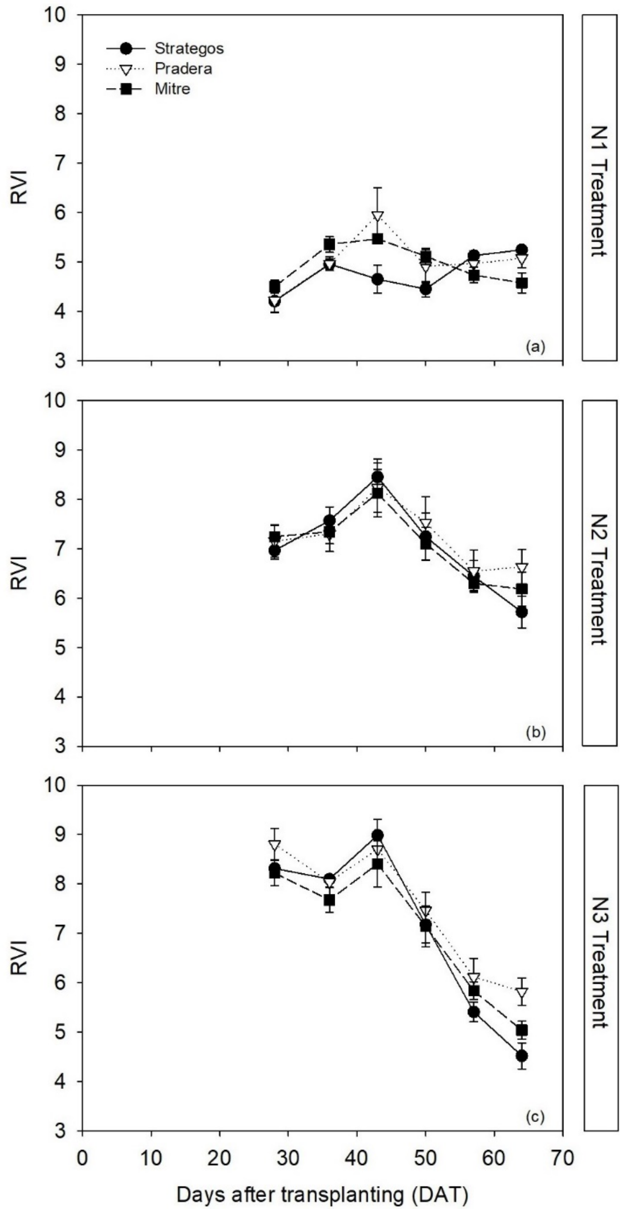

Figure S2. Temporal dynamics of RVI measurements of three cultivars of cucumber (*Cucumis sativus* L. 'Strategos', 'Pradera' and 'Mitre') under three N treatments (N1, N2 and N3). Values are means ± SE.

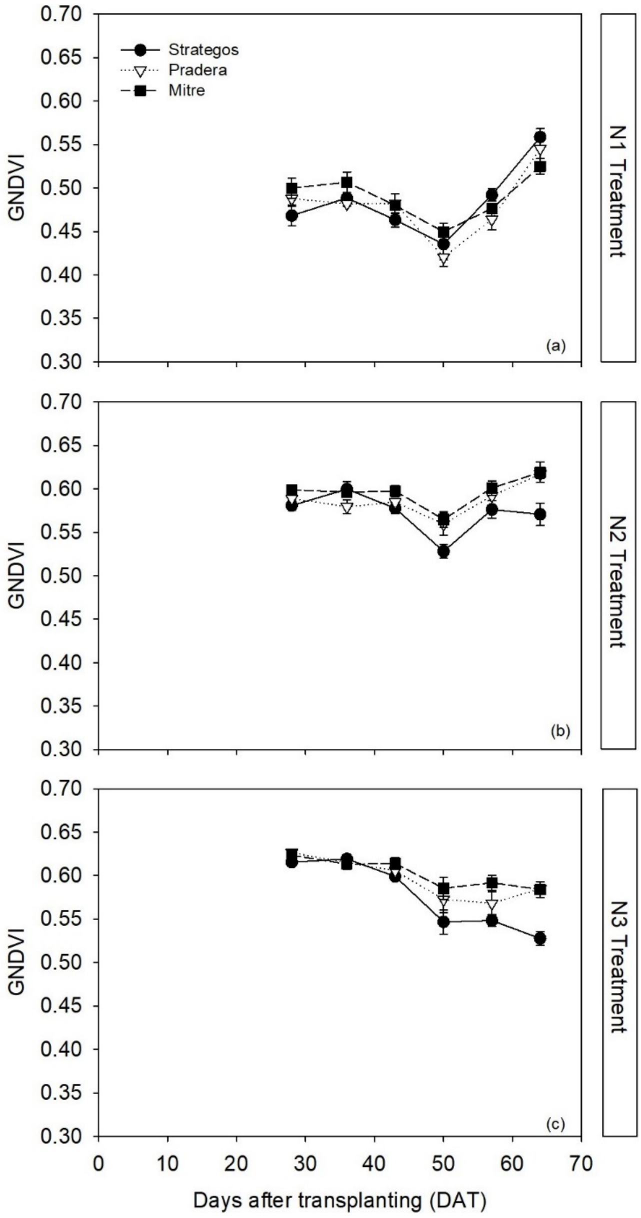

Figure S3. Temporal dynamics of GNDVI measurements of three cultivars of cucumber (*Cucumis sativus* L. 'Strategos', 'Pradera' and 'Mitre') under three N treatments (N1, N2 and N3). Values are means ± SE.
